# Analysis of Vehicle Detection with WSN-Based Ultrasonic Sensors

**DOI:** 10.3390/s140814050

**Published:** 2014-08-04

**Authors:** Youngtae. Jo, Inbum. Jung

**Affiliations:** Department of Computer Information and Communication Engineering, Kangwon National University, Chuncheon, Gangwondo 200-701, Korea; E-Mail: ytjoe@kangwon.ac.kr

**Keywords:** power saving, low computational complexity, ultrasonic sensors, traffic information acquisition systems, wireless sensor networks

## Abstract

Existing traffic information acquisition systems suffer from high cost and low scalability. To address these problems, the application of wireless sensor networks (WSNs) has been studied, as WSN-based systems are highly scalable and have a low cost of installing and replacing the systems. Magnetic, acoustic and accelerometer sensors have been considered for WSN-based traffic surveillance, but the use of ultrasonic sensors has not been studied. The limitations of WSN-based systems make it necessary to employ power saving methods and vehicle detection algorithms with low computational complexity. In this paper, we model and analyze optimal power saving methodologies for an ultrasonic sensor and present a computationally-efficient vehicle detection algorithm using ultrasonic data. The proposed methodologies are implemented and evaluated with a tiny microprocessor on real roads. The evaluation results show that the low computational complexity of our algorithm does not compromise the accuracy of vehicle detection.

## 1. Introduction

A wireless sensor network (WSN) is a large group of independent wireless sensor nodes comprised of sensors, microprocessors and communication modules. The objective of the WSN is to collect specific data and transmit it to the required destination. WSNs were originally studied for military purposes, but research is now focused on a wide range of consumer industries, giving rise to the notion of ubiquitous computing. Recently, new WSN approaches have been employed for more efficient traffic surveillance in intelligent transportation systems (ITS). Existing traffic information acquisition systems use wired power and communication and require powerful computing resources to attain high detection accuracy. However, such methodologies elevate the cost of construction and maintenance; consequently, the available detection area is narrowed. Thus, to measure traffic information across entire road networks, we require a low cost, highly scalable detection system.

WSN-based traffic information acquisition systems satisfy these twin requirements of low cost and high scalability. However, a number of issues must be overcome before WSN techniques are applied to traffic information acquisition systems. As the sensor nodes in WSNs are typically very small and driven by batteries, their computing power is limited. Thus, it is important to minimize power consumption, and to reduce as much as possible the computational complexity and memory usage of the vehicle detection algorithm. Studies on the application of WSNs to traffic surveillance have considered magnetic, accelerometer and acoustic sensors. However, ultrasonic sensors have not been applied in such a WSN environment.

Thus, in this paper, we introduce a power saving methodology for ultrasonic sensors and a low-complexity vehicle detection algorithm. Our approach does not compromise the vehicle detection accuracy. First, we analyze the characteristics of ultrasonic waves and sensors and then describe the vehicle detection methodology. Next, we discuss the power consumption of an ultrasonic sensor and provide detailed power-saving strategies. In addition, we introduce a novel detection algorithm that has low computational complexity and memory usage, while still providing an appropriate level of performance. The proposed vehicle detection algorithm is evaluated on real roads. Using the evaluation results, we discuss the possibilities and limitations of WSN-based traffic information acquisition systems with ultrasonic sensors.

The remainder of this paper is organized as follows. In the next section, we review the background to traffic information acquisition systems and the related literature. In Section 3, we describe the characteristics of ultrasonic waves, and in Section 4, we analyze and model the process of vehicle detection using WSN-based ultrasonic sensors. Section 5 describes a case study, and Section 6 presents the results of a performance evaluation. Finally, our conclusions and ideas for future work regarding this research are given in Section 7.

## 2. Related Work

Various sensors have been considered for the acquisition of traffic information in ITS. Existing systems can be considered as either intrusive or non-intrusive, depending on the sensor position. Intrusive sensors are installed under or across the pavement. They provide accurate traffic information, but the installation and maintenance of these sensors can cause traffic disruption. Examples of intrusive sensors include inductive loop detectors, pneumatic tubes, piezoelectric sensors and weigh-in-motion [1–4]. Non-intrusive sensors are installed above or to the side of the road, ensuring minimal disruption to traffic flow. Such sensors may utilize microwave radar, infrared, video, ultrasonic systems and acoustic sensors [5–9]. Existing systems collect highly accurate traffic information, but require high computing power, wired power supplies and wired communication. This increases their cost and decreases scalability.

To address these problems, traffic information acquisition systems based on WSNs have been studied. WSN-based systems use a wireless network and are battery driven, which enhances the system scalability and reduces the cost. In [10], a WSN-based vehicle detection system was introduced that used small magnetic sensors to replace conventional inductive loop detectors. Traffic flow and speed information could be collected with high accuracy, but this system did not allow for vehicle classification. Accurate vehicle classification was achieved by applying both magnetic and accelerometer sensors [11], where each vehicle could be detected by magnetic sensors and classified by axle detection using accelerometer sensors. A detection system has been proposed that uses acoustic sensors to provide wide-range, low-cost traffic surveillance [12]. This system collects speed and flow data at a master node, and traffic congestion is detected by the sensor nodes. Pyro-electric sensors were employed to detect vehicles in a parking space and a typical road environment [13,14]. In [13], pyro-electric sensors were used to sense the status of car parking space. The status information is displayed for the users on the LED (Light-Emitting Diode) screen. The authors in [14] tried to increase vehicle detection accuracy by combining different type of sensors, such as acoustic, magnetic, accelerometer and pyro-electric sensors.

The WSN-based systems mentioned above have proved that low-cost, highly scalable traffic surveillance can be accomplished. However, many issues remain to be overcome. The approaches in [10,11] showed that vehicle detection and classification can be achieved using multimodal sensor data, but these systems should be installed on the pavement, which will increase their cost and cause traffic disruption. The approach reported in [12] is non-intrusive, but could not achieve high detection accuracy across multiple lanes. The studies in [13,14] are also non-intrusive, but did not provide detailed methods of power saving and lightweight detection algorithm.

**Figure 1.**
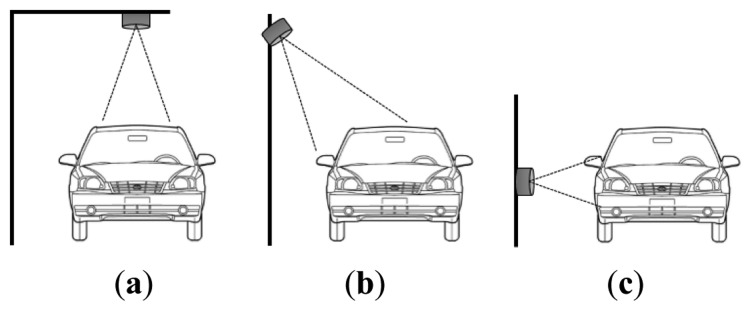
Mount positions of an ultrasonic sensor. (**a**) Overhead mount; (**b**) side top mount; (**c**) horizontal mount.

In our previous paper, we introduced entire architecture of vehicle detection systems with ultrasonic sensors in WSN [15]. However, the detailed methodologies of power saving and lightweight detection algorithm were not provided. Thus, in this paper, we propose advanced methods of power saving and lightweight detection algorithm using WSN-based ultrasonic sensors. Figure 1 illustrates some typical mount positions for ultrasonic sensors. We use the horizontal mount position shown in Figure 1c, as this enhances the scalability of the proposed system.

## 3. Characteristics of Ultrasonic Waves

This section describes the characteristics of ultrasonic waves. Generally, frequencies above 20 kHz are considered to be ultrasonic. The speed of ultrasonic waves varies with air temperature and can be calculated as follows:

(1) Ultrasonic speed=331.5m/s+(0.61×temperature)

**Figure 2.**
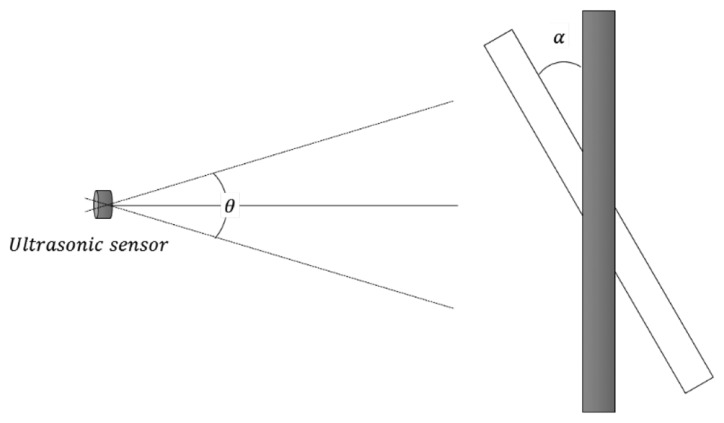
Dissemination and detectable angle of an ultrasonic sensor.

The dissemination angle of an ultrasonic sensor depends on the ultrasonic frequency. As illustrated in Figure 2, for instance, the dissemination angle θ increases as the ultrasonic frequency decreases. In addition, to detect the reflected ultrasonic wave, we require $\alpha <\frac{\theta }{2}$, where α is the angle between an ultrasonic sensor and the detection object.

These characteristics of ultrasonic waves must be considered when designing vehicle detection systems. The variation of ultrasonic speed with air temperature influences the detection interval of an ultrasonic sensor, and the angle between the sensor and detection object influences its installation position. These factors are discussed further in Section 4.

## 4. Vehicle Detection Using Ultrasonic Sensors

**Figure 3.**
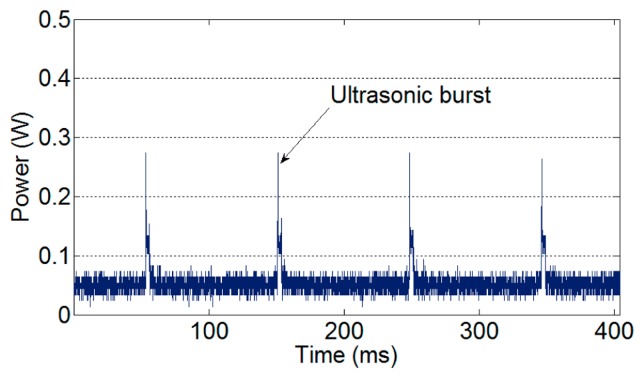
Power consumption of an ultrasonic sensor.

The important aspect of WSN-based traffic information acquisition systems is to minimize power consumption. Because WSN-based systems are battery driven, their total lifetime is restricted by the residual battery capacity. Typically, the largest power consumption occurs with ultrasonic bursts in an ultrasonic sensor. Figure 3 shows the power consumption of an ultrasonic sensor under a series of ultrasonic bursts.

**Figure 4.**
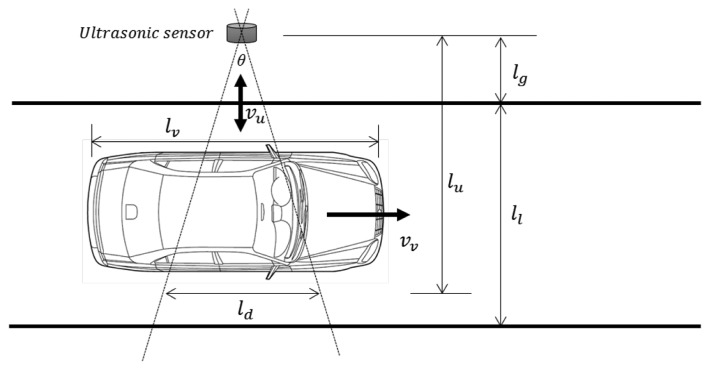
Analysis model.

Thus, minimizing the number of ultrasonic bursts will reduce the power consumption of the sensor. Using a long detection interval can reduce the power consumption caused by such bursts, but an overly long detection interval will increase the number of detection errors. This is because a certain amount of data is needed to guarantee accurate vehicle detection. Therefore, it is important to determine an appropriate detection interval for WSN-based traffic information acquisition systems. To do this, we constructed the analysis model illustrated in Figure 4.

### 4.1. Analysis with a Single Vehicle

First, to simplify our analysis, we consider a single vehicle on a single-lane road, as illustrated in Figure 4. We assume the vehicle has length *l_v_* and is traveling at constant speed *v_v_* on a straight path along a lane of width *l_l_*. The ultrasonic sensor is placed at a distance of *l_g_* from the roadside. The ultrasonic wave spreads at constant speed *v_u_* and with angle θ. The width of the ultrasonic wave *l_d_* is $2l_{u}\tan\frac{\theta }{2}$, where *l_u_* is the distance from the sensor to the detection point.

The detection point is the side of the vehicle owing to the mount position of an ultrasonic sensor. Vehicles are detected using a number of distance data collected from the ultrasonic sensors. This requires the ultrasonic sensors to have detection intervals that can collect sufficient distance data for reliable vehicle detection.

The detection interval of an ultrasonic sensor is given by:

$$\frac{\text{detectable period of a vehicle}}{\text{number of distance data necessary for vehicle detection}}$$

In Figure 4, the detectable period of the vehicle can be calculated as $\frac{l_{v}}{v_{v}}$ when the vehicle passes the ultrasonic sensor. However, as the ultrasonic wave has a width of *l_d_* at detection distance *l_l_*, the detectable period of the vehicle is $\frac{l_{v}+l_{d}}{v_{v}}$. Thus, if we know how many distance measurements are needed, we can calculate the detection interval. However, this is not simple. We must examine the system design to determine the necessary number of distance data.

Generally, raw signal data collected from the sensors should be filtered for noise before being input to a specific algorithm. To avoid useful signals being filtered out, the volume of data should be larger than some minimum amount required by the noise filter. This minimum varies depending on the type of noise filter. The most appropriate noise filter is determined by the characteristics of ultrasonic sensors.

Ultrasonic sensors generate distance data by measuring the time taken to receive reflected ultrasonic waves. The time is directly proportional to the distance between the ultrasonic sensor and the detection object. Owing to the slow speed of ultrasonic waves, the sampling rate is much lower than in other sensors, e.g., acoustic and magnetic sensors. Moreover, if the noise filter is implemented in a software package, its complexity must be suitable for the low computing power of WSN-based systems. Considering these two restrictions, we analyzed various filters. A median filter has been selected for the proposed system, because it combines appropriate performance with a low sampling rate.

The required number of data for a median filter is $\left\lceil \frac{m}{2}\right\rceil$, where *m* is the mask size of the filter. Thus, the detection interval of an ultrasonic sensor using a median filter is given by Equation (2), which means the maximum detection interval. If the detection interval is longer than that given by Equation (2), the data will be filtered out:

(2) $$\text{Maximum detection interval}=\frac{l_{v}+l_{d}}{v_{v}\left\lceil \frac{m}{2}\right\rceil }$$

Previously, the maximum detection interval for an ultrasonic sensor was derived based on the model in Figure 4. If more frequent data are needed, a shorter detection interval can be applied. However, the detection interval cannot be shortened indefinitely, owing to the slow speed of ultrasonic waves. If a new ultrasonic wave is emitted before the previous wave has been received, a detection error will occur. Thus, the ultrasonic sensor should wait until the previous wave has arrived.

In Figure 4, the maximum detection distance is *l_l_* + *l_g_*. Because the ultrasonic wave makes a round trip, the maximum total distance traveled by the ultrasonic wave is 2(*l_l_* + *l_g_*). If a road has *n* lanes, this distance will be 2(*nl_l_* + *l_g_*). The travel time of an ultrasonic wave over this distance is given by Equation (3). This is the minimum detection interval, because the ultrasonic sensor cannot emit a new ultrasonic wave during this period:

(3) $$\text{Minimum detection interval}=\frac{2\left(nl_{l}+l_{g}\right)}{v_{u}}$$

Thus, the available detection interval *t_i_* can be written as:

(4) $$\text{Available detection interval}\to \frac{2\left(nl_{l}+l_{g}\right)}{v_{u}}<t_{i}<\frac{l_{v}+l_{d}}{v_{v}\left\lceil \frac{m}{2}\right\rceil }$$

If the detection interval of an ultrasonic sensor is outside these bounds, the reliability of the collected data cannot be guaranteed.

We can now determine an appropriate detection interval for an ultrasonic sensor in a road environment. If power saving is more important, a longer detection interval can be applied, whereas a shorter interval can be used when sufficient power resources are available. This shorter detection interval will provide more distance data. However, the parameters of Equation (4) vary depending on the road environment. Thus, further analysis is necessary.

**Figure 5.**
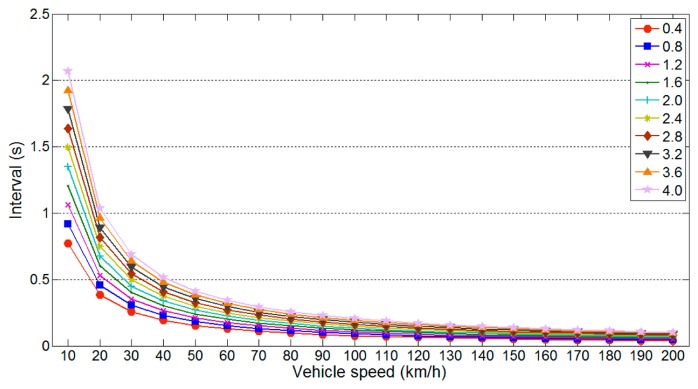
Variation in the maximum detection interval.

According to Equation (2), the vehicle speed *v_v_* and detection distance *l_u_* affect the maximum detection interval (*l_d_* varies according to *l_u_*). Figure 5 illustrates the variation in the maximum detection interval with *v_v_* for different values of *l_u_*. In this simulation, we used parameter values of *m* = 3, *l_v_* = 3.5 m and θ = 90. As shown in Figure 5, the maximum detection interval decreases linearly with shorter *l_u_*, whereas it decreases logarithmically with higher *v_v_*.

**Figure 6.**
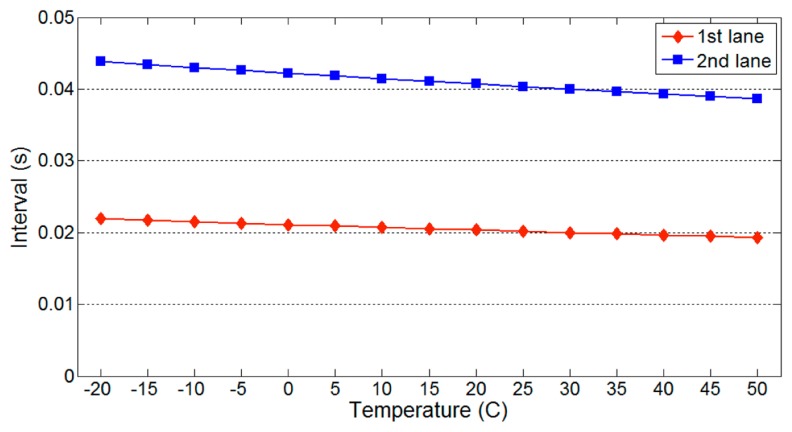
Variation in the minimum detection interval.

According to Equation (3), the distance traveled by an ultrasonic wave and the ultrasonic speed *v_u_* influence the minimum detection interval. Figure 6 illustrates the variation in the minimum detection interval with temperature for different numbers of lanes. Parameter values of *l_l_* = 3.5 m and *l_g_* = 0 were used. As expected, the minimum detection interval decreases for shorter travel distances. Because the speed of an ultrasonic wave increases with temperature, the minimum detection interval also decreases with higher temperature. However, the variation with temperature is insignificant and is thus neglected in our later analysis.

Figures 5 and 6 show that the maximum and minimum detection intervals are affected by specific parameters. Among these, *l_u_* significantly influences both detection intervals. Therefore, it is necessary to determine the detection interval that is least affected by variations in this parameter.

**Figure 7.**
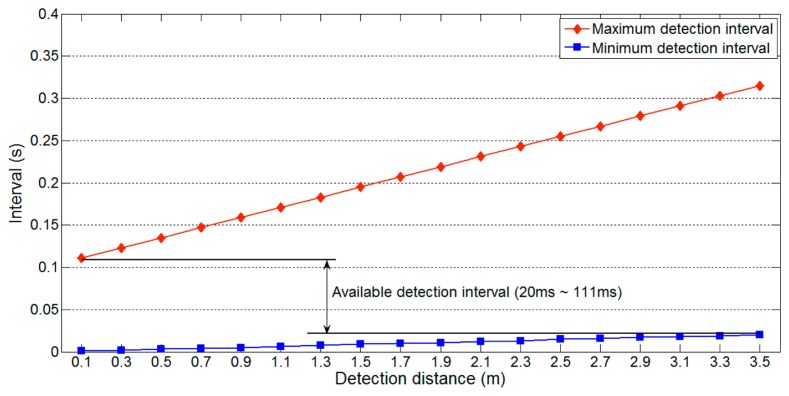
Available detection interval on a one-lane road.

Figure 7 shows the maximum and minimum detection interval on a one-lane road with respect to the detection distance *l_u_*, maximum and minimum detection intervals vary significantly with the detection distance. Thus, for reliable vehicle detection, the available detection interval should be from 20 ms to 111 ms, the interval that is not affected by the variation in detection distance. Because Equation (3) uses the maximum value of *l_u_* (*i.e.*, 2(*nl_l_* + *l_g_*)), the minimum detection interval is not influenced by changes in *l_u_*. However, Equation (2) varies with *l_u_*, so Equation (4) should include the following additional restrictions:

(5) $$\text{Available detection interval}\to \frac{2\left(nl_{l}+l_{g}\right)}{v_{u}}<t_{i}<\frac{l_{v}+l_{d}}{v_{v}\left\lceil \frac{m}{2}\right\rceil }$$

*l_d_* = width of minimum *l_u_*;
*l_v_* = minimum vehicle length;
*v_v_* = maximum vehicle speed.

First, for the maximum detection interval, the minimum value of *l_d_* (*i.e.*, the width given by the minimum *l_u_*) should be used, because Figure 7 indicates that larger values lead to unreliable vehicle detection. The minimum of *l_d_* varies according to the sensor position and road environment, but this information can be easily measured. Second, the vehicle length *l_v_* should be minimized. Thus, we may use the length of a typical compact car. Finally, the vehicle speed *v_v_* should be the maximum speed on the target road. The speed limit, or a slightly larger value, can be used for *v_v_*, as people often drive above the speed limit [16]. The main reason for these restrictions on *l_v_* and *v_v_* is that they affect the maximum detection interval.

**Figure 8.**
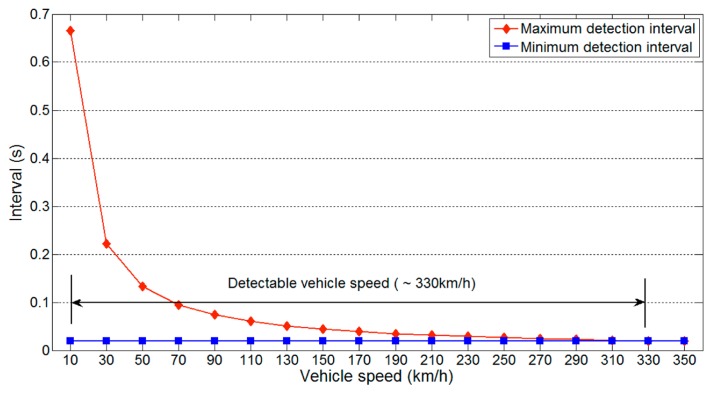
Detectable vehicle speed on a one-lane road.

As we have mentioned, the sampling rate of an ultrasonic sensor is much lower than that of other sensors. Thus, the detectable vehicle speed represents important information. As expected, the detectable vehicle speed is also lower than with other sensors, owing to the slow sampling rate of ultrasonic sensors. Figure 8 shows the detectable vehicle speed on a one-lane road using parameter values of θ = 90, *m* = 3, *l_v_* = 3.5 m, *l_d_* = 0.2 m, *l_l_* = 3.5 m, *l_g_* = 0 m and *v_u_* = 343.7m/s.

Because the minimum detection interval is not influenced by the vehicle speed, this remains constant for different vehicle speeds. In contrast, the maximum detection interval decreases with higher vehicle speeds. The two values meet at about 330km/h, which is the maximum detectable vehicle speed. This speed is more than sufficient for a normal one-lane road, indicating that reliable data collection on such roads is possible.

**Figure 9.**
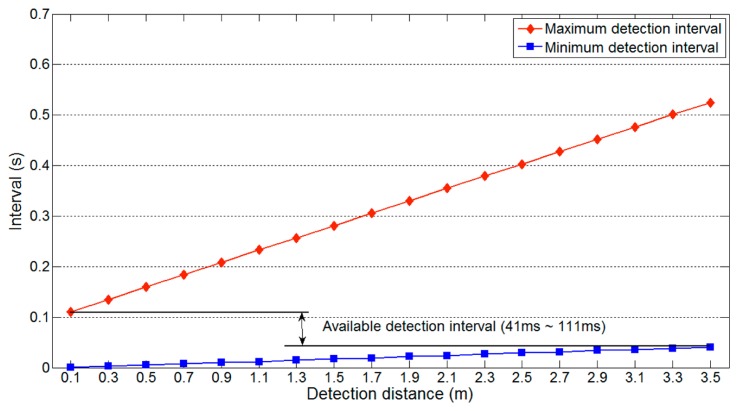
Available detection interval on a two-lane road.

**Figure 10.**
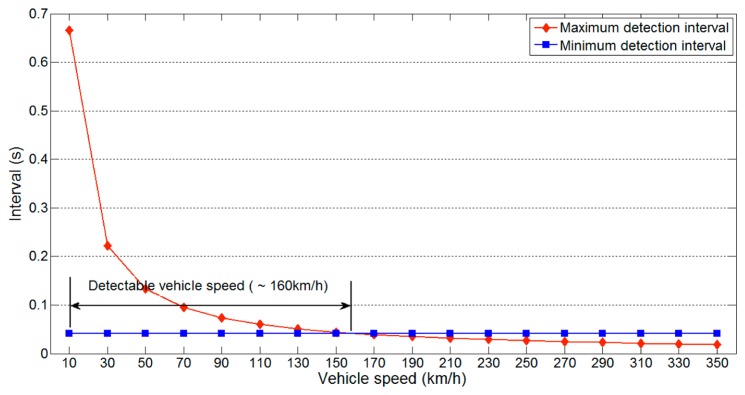
Detectable vehicle speed on a two-lane road.

The same experiments were performed on a two-lane road, and the results are shown in Figures 9 and 10. The same parameters as used to produce Figures 7 and 8 were used. Figure 9 shows that the available detection interval is narrower than for a one-lane road, because the longest minimum detection interval increases from 20 ms to 41 ms. This increase is a result of the increase in the detection distance. The detectable vehicle speed is also reduced owing to the increase in minimum detection interval. These results indicate that more lanes reduce the available detection interval and detectable vehicle speed.

### 4.2. Analysis with Multiple Vehicles

**Figure 11.**
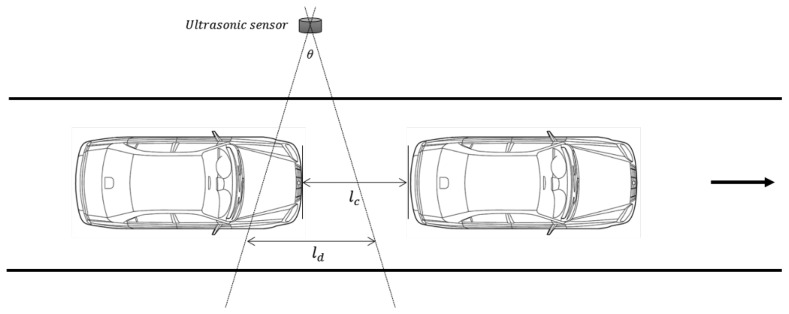
Analysis model with multiple vehicles.

The previous analysis of the available detection interval considered a single vehicle. We now extend our analysis to a multiple-vehicle environment. Figure 11 illustrates the analysis model with multiple vehicles. For reliable vehicle detection with more than two vehicles, it is vital that two vehicles can be distinguished. To separate the two vehicles, a certain amount of data should be measured in the space between the two vehicles. This means that $t_{g}>\left\lceil \frac{m}{2}\right\rceil t_{i}$, where *t_g_* is the temporal gap between the vehicles, *m* is the mask size of the median filter and *t_i_* is the detection interval of an ultrasonic sensor. Moreover, $l_{c}>l_{d}+\left\lceil \frac{m}{2}\right\rceil t_{i}v_{v}$ should hold, where *l_c_* is the distance between two vehicles. These restrictions can be summarized as:

(6) $$\text{Requirements to separate two vehicles}\to \{\begin{array}{c} t_{g}>\left\lceil \frac{m}{2}\right\rceil t_{i}\\ l_{c}>l_{d}+\left\lceil \frac{m}{2}\right\rceil t_{i}v_{v} \end{array}$$

The first constraint of Equation (6) implies that, if *t_g_* is shorter than $\left\lceil \frac{m}{2}\right\rceil t_{i}$, the collected data will be filtered out by the noise filter. As a result, the two vehicles cannot be separated. In the second constraint, *l_c_* > *l_d_* must be satisfied. If *l_c_* < *l_d_*, the ultrasonic sensor cannot collect any data from between the two vehicles. There must be sufficient data, after filtering, from between the two vehicles, which requires additional space between them. Thus, the distance moved by the vehicle at the rear during the total detection period should be added to *l_d_*. The total detection period is $\left\lceil \frac{m}{2}\right\rceil t_{i}$, so the distance moved by the rear vehicle is $\left\lceil \frac{m}{2}\right\rceil t_{i}v_{v}$.

We now describe how Equation (6) affects our previous analysis. First, we must calculate the gap time *t_g_* to analyze the first requirement of Equation (6). However, this gap time varies according to vehicle speed and road environment, so we cannot apply a constant gap time for the first requirement of Equation (6).

**Figure 12.**
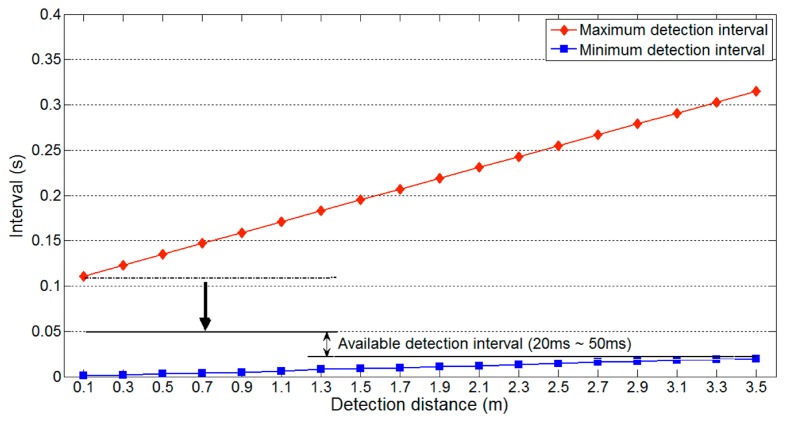
Available detection interval on a one-lane road.

Thus, we consider a specific gap time to explain the influence of Equation (6). Figure 12 shows the available detection interval from Figure 7 adjusted by the first requirement of Equation (6) with parameter values of *t_g_* = 0.1 s and *m* = 3. As the gap time is assumed to be 0.1 s, the constraint imposed by Equation (6) causes the maximum detection interval to decrease from 111 ms to 50 ms. Although we assumed a gap time of 0.1 s, this will typically be longer on real roads. If a longer gap time was used for the previous analysis, the available detection interval would be unchanged. For example, if the gap time is longer than 222 ms, it does not influence the available detection interval. Thus, an appropriate estimate of the gap time is important to obtain a reliable detection interval.

We now consider the second requirement of Equation (6). If the angle θ of the ultrasonic sensor is reduced, the width of the ultrasonic wave *l_d_* decreases, which means that the minimum distance required between two vehicles can be reduced by a narrower ultrasonic angle. Thus, the second constraint of Equation (6) implies that a narrower angle in the ultrasonic sensor will enable easier separation of two vehicles.

### 4.3. Other Considerations

The previous analysis results demonstrate the importance of determining the detection interval for power saving in WSN-based traffic information acquisition systems that use ultrasonic sensors. In this section, we discuss other considerations that influence the detection interval.

**Figure 13.**
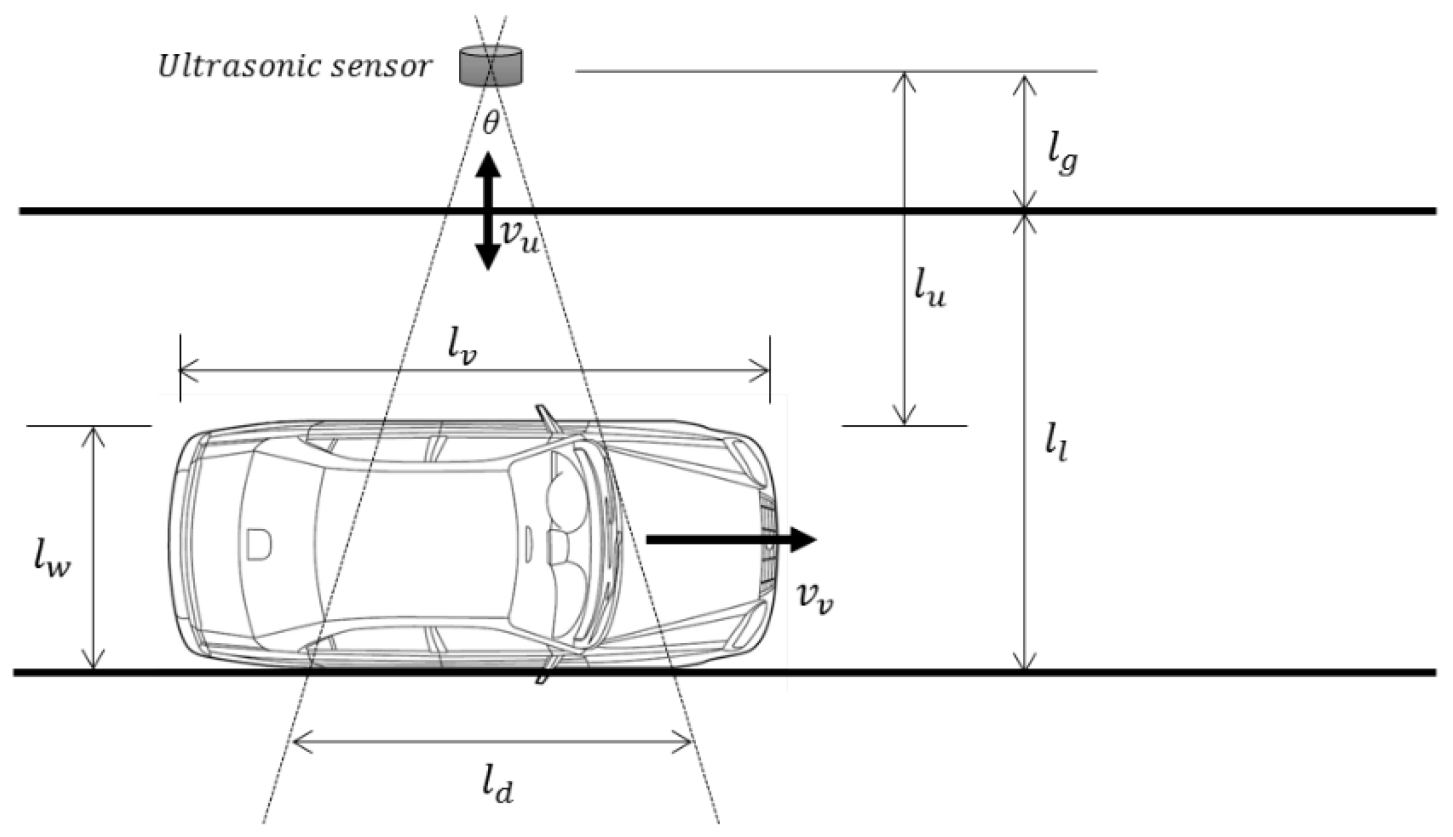
Modified analysis model.

Figure 13 illustrates a modified analysis model. When a vehicle passes the ultrasonic sensor, the maximum value of the detection distance *l_u_* cannot exceed *l_l_* + *l_g_* − *l_w_*, where *l_w_* is the width of the vehicle. Thus, we have that *l_u_* <*l_l_* + *l_g_* − *l_w_*, *i.e.*,

(7) $$0<l_{u}<l_{l}+l_{g}-l_{w}$$

**Figure 14.**
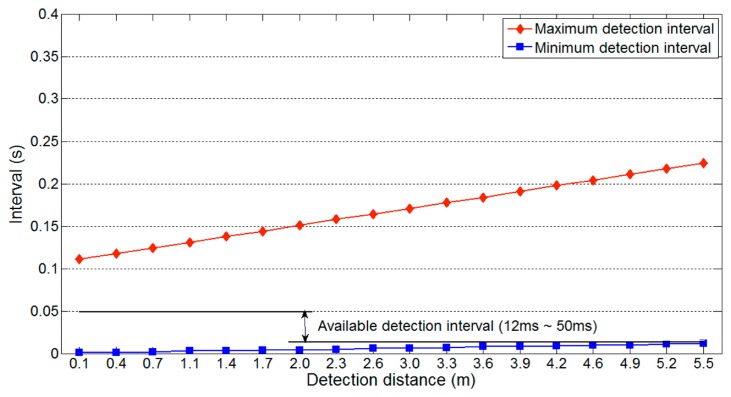
Available detection interval on a one-lane road.

Equation (7) implies that reducing the maximum value of *l_u_* decreases the longest possible minimum detection interval. Figure 14 shows the effect of Equation (7) on the available detection interval in Figure 12. The parameters used are the same as those used to produce Figure 12, and *l_w_* = 1.5 m (the approximate width of a compact car). As shown in Figure 14, the constraints of Equation (6) mean that the maximum detection interval is fixed to 50 ms. In contrast, the longest minimum detection interval decreases from 20 ms to 12 ms. This reduction widens the range of the available detection interval, which indicates that data can be collected more frequently and increases the detectable vehicle speed.

**Figure 15.**
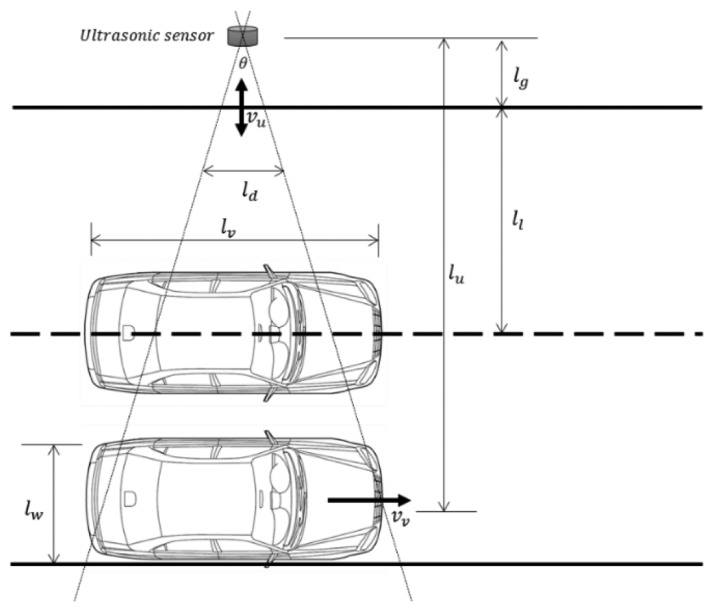
Modified analysis model on a two-lane road.

The analysis model of Figure 13 considered a one-lane road. The model can be extended to a two-lane road, as illustrated in Figure 15, in which one vehicle is placed in each lane. In this case, the maximum value of *l_u_* for the vehicle in the inside (respectively outside) lane will be 2*l_l_* + *l_g_* − *l_w_* (respectively $l_{l}+l_{g}-\frac{l_{w}}{2}$). These two conditions can be summarized as follows:

(8) $$\begin{array}{c} 0<l_{u}<l_{l}+l_{g}-\frac{l_{w}}{2}\\ l_{l}+l_{g}-\frac{l_{w}}{2}<l_{u}<2l_{l}+l_{g}-l_{w} \end{array}$$

Equation (8) also influences the range of the available detection interval, and the impact on the longest minimum detection interval is the same as that resulting from Equation (7).

**Figure 16.**
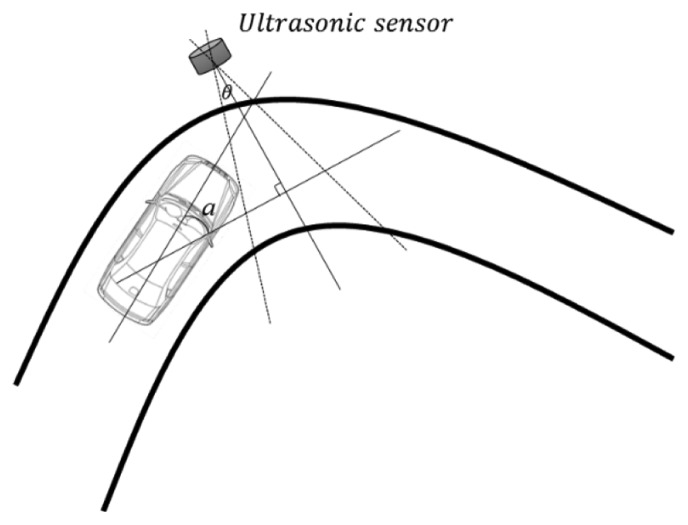
Ultrasonic sensor positioning.

Finally, we discuss the positioning of the ultrasonic sensors. As shown in Figure 2, we require $\alpha <\frac{\theta }{2}$ to correctly detect vehicles, and this condition influences the positioning of the ultrasonic sensors. Figure 16 illustrates that, if an ultrasonic sensor is placed on a curved section of road, the value of α will increase. Consequently, the reflected ultrasonic wave cannot be received by the ultrasonic sensor. Thus, ultrasonic sensors should be installed on straight sections of road to ensure reliable vehicle detection.

## 5. Case Study

In this section, we use a case study to introduce a framework for designing a lightweight vehicle detection algorithm. The target road has two lanes and an 80 km/h speed limit. Initially, we use the detection interval derived from our previous analysis and then explain the vehicle detection algorithm.

### 5.1. Detection Interval

**Figure 17.**
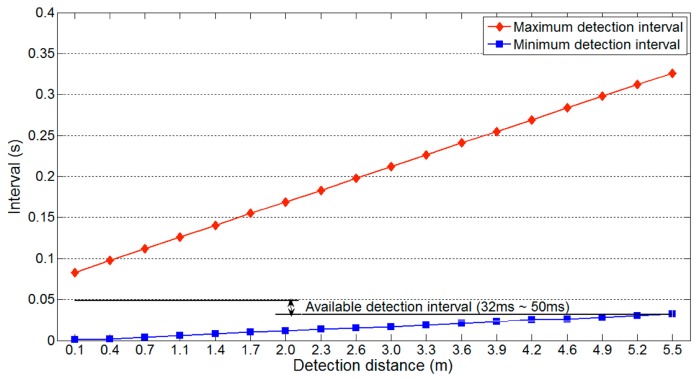
Available detection interval on a two-lane road.

The parameters of the target road are *n* = 2, θ = 90, *m* = 3, *l_v_* = 3.5 m, *l_l_* = 3.5 m, *l_g_* = 0 m, *v_v_* = 80 km/h and *v_u_* = 343.7 m/s. The available detection interval of 32 ms to 50 ms is generated using Equations (5), (6) and (8) (Figure 17). As WSN-based systems should generally minimize their power consumption, we select the longest available detection interval.

### 5.2. Vehicle Detection Algorithm

**Figure 18.**
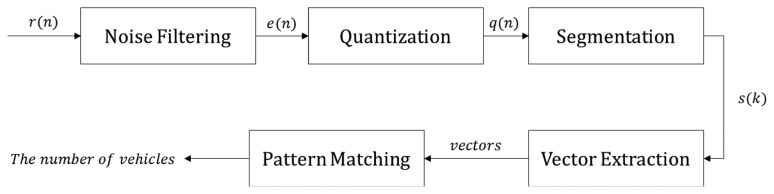
Vehicle detection algorithm.

Figure 18 depicts an overview of our vehicle detection algorithm. As WSN-based systems have low computing power, algorithms with high computational complexity cannot be applied. Moreover, memory reduction mechanisms should be included owing to the limited memory size. Our algorithm is designed based on these restrictions.

Our algorithm is classified into five steps. The raw distance data *r*(*n*) measured by an ultrasonic sensor are passed through a noise filter. We use a median filter, because this retains good performance with the low sampling rate of ultrasonic data. To minimize the computational complexity of the median filter, a mask size of three is used. The filtered distance data *e*(*n*) are then quantized according to the lane width. This step is very important, because it limits the type of vectors to be handled in the vector extraction step. This, in turn, minimizes the search overhead in the pattern matching step. Moreover, quantization simplifies the segmentation operations.

**Figure 19.**
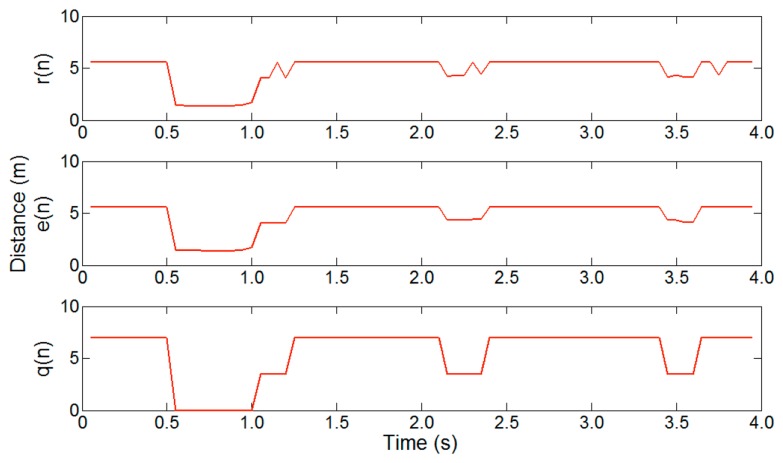
Example of noise filtering and quantization.

Figure 19 shows the effect of noise filtering and quantization on a two-lane road, where *r*(*n*) is raw distance data, *e*(*n*) is noise filtered data and *q*(*n*) is quantized data, with a mask size of three and a quantization level of 3.5 m. From the filtered data *e*(*n*), we can confirm that the median filter exhibits proper filtering performance with ultrasonic data. The quantized data *q*(*n*) clearly show information about the passing vehicles.

**Figure 20.**
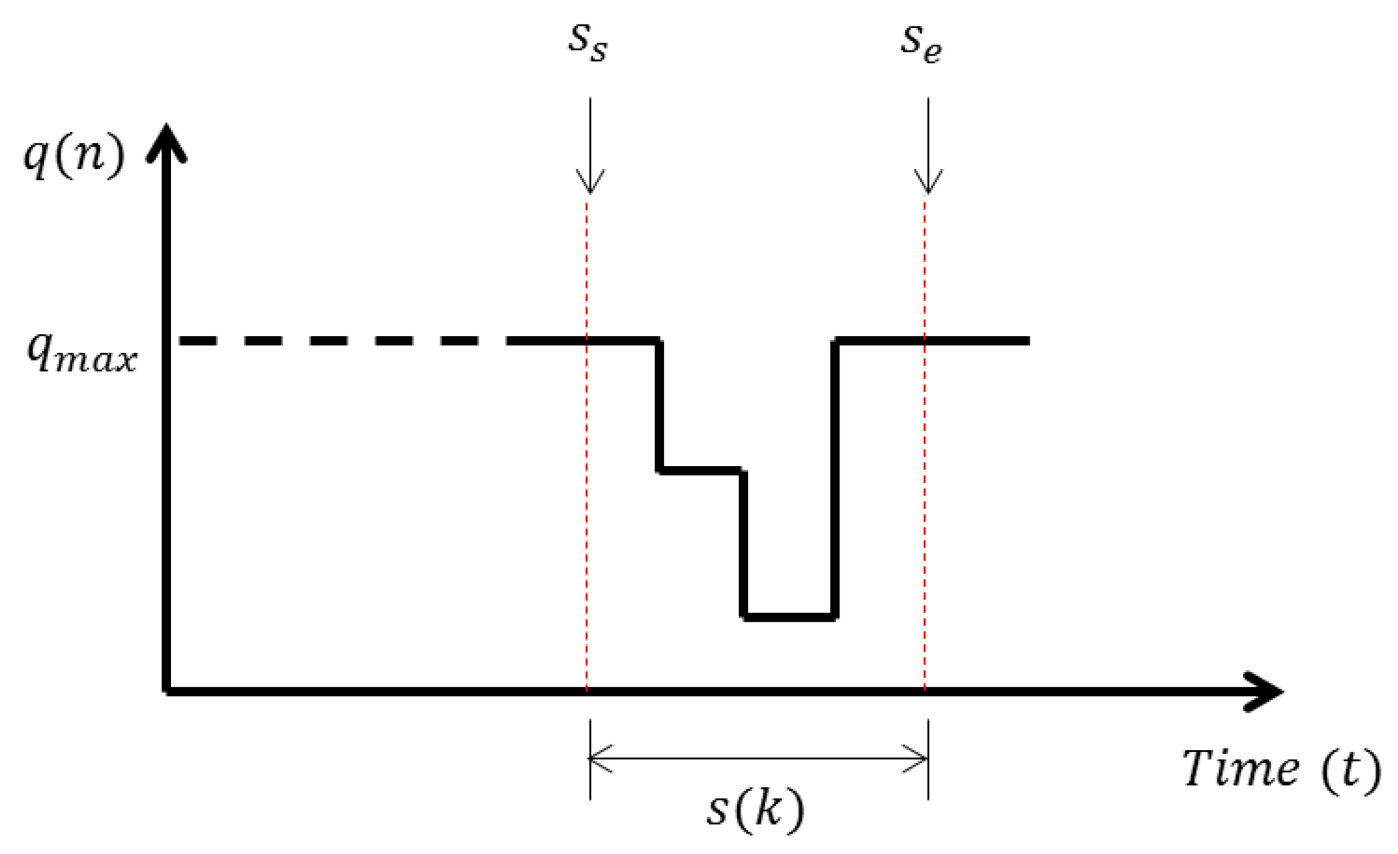
Segmentation.

As shown in Figure 20, if the specific data that includes traffic information can be segmented, we can greatly reduce the amount of memory needed. The starting point *s_s_* and end point *s_e_* of segmentation can be easily selected as:

(9) $$\begin{array}{c} s_{s}=q\left(t\right),\;\text{if}\;q\left(t\right)<q_{\text{max}}\\ s_{e}=q\left(t\right),\;\text{if}\;q_{\text{max}}\;\text{and}\;s_{s}\;\text{is enabled} \end{array}$$

where *q_max_* is the maximum distance data measured by an ultrasonic sensor, which means that there are no vehicles on roads where *q*(*t*) is the quantized distance data at time *t*. This simple method can be applied owing to the previous quantization step. More complicated mechanisms would be needed to select *s_s_* and *s_e_* without the quantization.

After quantization, a segmentation process is employed to minimize the amount of memory required. The periodic collection of traffic information is widely used in traffic information acquisition systems. For instance, the intelligent roadway information system in Minneapolis, Minnesota, USA, collects traffic information every 30 s [17]. If two bytes of distance data are collected at 50 ms intervals, a total of 1.2 KB will be generated every 30 s. The widely used WSN platform MICAz (Crossbow, Milpitas, CA, USA) has 4 KB memory space [18]. Thus, considering that the WSN computing platform must run a routing protocol and operating system at the same time, 1.2 KB is a significant amount of data.

**Figure 21.**
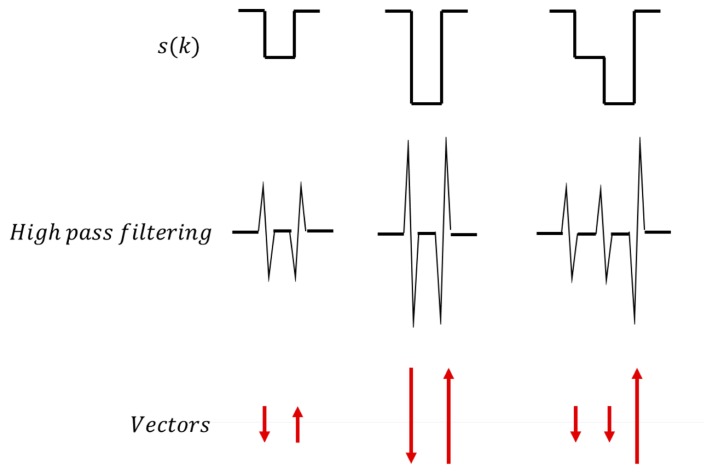
Vector extraction.

**Figure 22.**
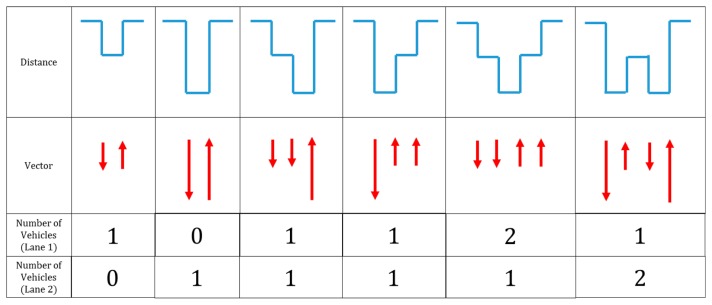
Example patterns for the target road.

The segmented distance data are then represented as vectors, as depicted in Figure 21. To change the segmented distance data *s*(*k*) to vectors, a high-pass filter is applied. As shown in Figure 21, high-pass-filtered data have their own size and direction, depending on the quantized distance data. The quantization according to lane width in the previous step limits the possible vector types. For a two-lane road, only four different vectors can be generated (two different directions and two different sizes). Figure 22 illustrates example vectors and patterns for our target road. Each pattern represents the number of lanes on the road. There are a total of six patterns for our target road. Using these patterns, traffic information is generated in the final pattern matching step.

### 5.3. Vehicle Detector

**Figure 23.**
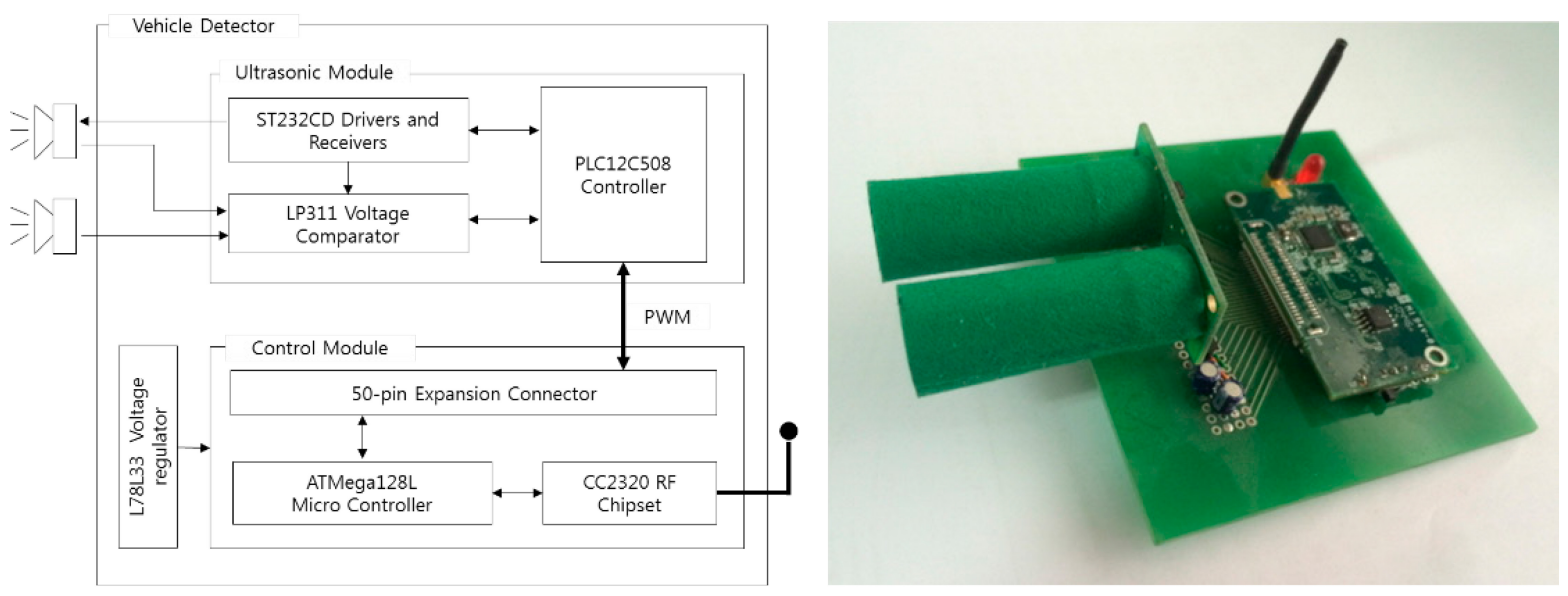
Vehicle detector.

Figure 23 shows the internals of our vehicle detector. The detector is composed of an ultrasonic module and a control module. We used an SRF04 (Devantech, Attleborough, England) from Devantech [19] for the ultrasonic module and a MICAz from Crossbow [18] for the control module. The ultrasonic module is controlled by a pulse-width modulation (PWM) signal generated by the Atmega128L (Atmel, San Jose, CA, USA) in the control module. The distance data measured by the ultrasonic module are transmitted to the control module as an analog signal, and then, the received distance data are interpreted as traffic information using our vehicle detection algorithm. The collected traffic information is transmitted to a server by a CC2420 radio transceiver. (Texas Instruments, Dallas, TX, USA) The software is based on TinyOS, which is the most popular WSN operating system [20].

## 6. Performance Evaluation

Our vehicle detection algorithm is designed to minimize both computational complexity and memory usage. We implemented our algorithm on the MICAz mote, which has only an 8 MHz clock speed and 4 KB of memory. We confirmed the complete operation of our algorithm with TinyOS in real time. It indicated that our algorithm is designed with greatly lowered computational complexity and memory usage.

Next, we analyzed the accuracy of our vehicle detection algorithm. The experiments considered a two-lane road from 10:00 to 11:00. Table 1 compares the detection results with video results.

In Table 1, the possible detection errors in our system are classified into three categories. An “overlap” error occurs when two vehicles in different lanes pass the detector at the same time. In this case, the vehicle furthest from the sensor cannot be detected. A “loss” error occurs when the vehicle detection data are filtered out by the noise filter. Finally, an “over-counting” error is caused by a vehicle lane change. If a vehicle changes lanes in front of the detector, the data pattern is measured as the third or fourth pattern in Figure 22. The “loss” and “over-counting” errors can occur in other types of sensors, such as magnetic and acoustic sensors. However, “overlap” errors are specific to the roadside installation of sensors. It is obvious that the number of “overlap” errors will increase with higher traffic flow.

As shown in Table 1, the total number of detection errors using the ultrasonic sensors is 6.37% lower in Lane 1 and 0.55% higher in Lane 2. This difference is because the detector is installed on the roadside next to Lane 2. The total error rate is 1.53% lower than that recorded by the video sensors, which indicates that our algorithm has high vehicle detection accuracy.

The previous analysis and performance evaluation indicate the feasibility of vehicle detection with ultrasonic sensors within a WSN-based traffic information acquisition system. The traffic information can be collected in real time with small, simple hardware and software. Moreover, the detection accuracy is very high. However, such a system has some limitations. The roadside installation of sensors leads to “overlap” errors, and their number will increase with higher traffic flows. Our detector was tested on a road with 522veh/h, which is a normal traffic flow on a two-lane road. If the experiments were performed with higher traffic volumes, the error rate would be higher than that reported here. This suggests that roadside sensor installation, while improving the system's scalability, can increase the number of detection errors.

Thus, to maintain detection accuracy, ultrasonic sensors should only be employed on roads with few lanes, and dense traffic flows should be avoided. These two limitations can be overcome with small changes. For instance, the detection accuracy could be increased across multiple lanes by installing sensors on both sides of the road. In addition, our system could be used for temporal vehicle detection, such as within a work zone.

It is true that the roadside installation of sensors has limitations for accurate vehicle detection with dense traffic flows. Other mount positions, such as overhead and side top mount, in Figure 1 can be used. In these case, the scalability will decline, because each lane needs its own sensor. However, our strategies for power saving and the framework for the lightweight vehicle detection algorithm introduced in this paper can be applied for the other mount positions.

## 7. Conclusions and Future Work

In this paper, we analyzed vehicle detection using roadside ultrasonic sensors in WSNs and suggested methodologies for power saving and lightweight vehicle detection. From the detailed analysis, we confirmed the importance of determining an appropriate detection interval in minimizing power consumption. We introduced design methodologies to reduce computational complexity and memory usage while maintaining high detection accuracy. However, our experiments showed that vehicle detection across multiple lanes with a single roadside ultrasonic sensor suffers a reduction in detection accuracy under dense traffic flow, because the number of “overlap” errors increases. Thus, to guarantee high detection accuracy, ultrasonic sensors should be employed in situations with few lanes and low traffic flows.

However, the two limitations can be overcome with some effort. For instance, installing sensors on both sides of the road reduces the detection complexity of a single ultrasonic sensor, which can increase vehicle detection accuracy with multiple lanes. Moreover, applying a narrower dissemination angle of an ultrasonic sensor can increase detection accuracy owing to easier separation of two vehicles.

Our detailed analysis and evaluation will provide a guide for the future development of traffic information acquisition systems using ultrasonic sensors.

We considered a single sensor for multiple lanes in this paper. In the future, we will apply multiple sensors across multiple lanes. This will require accurate time synchronization among the sensors. Thus, we will study the efficient time synchronization of multiple sensors in WSNs. Moreover, we will examine the use of other sensor types for WSN-based traffic information acquisition systems.

## Figures and Tables

**Table 1.** Vehicle detection results.

Time	Count by Video (vehicles)			Count by Ultrasonic (vehicles)			Error Rate (%)			Type of Error (vehicles)				
	
										Overlap	Loss		Over Counting	
	
	Lane 1	Lane 2	Total	Lane 1	Lane 2	Total	Lane 1	Lane 2	Total	Lane 1	Lane 1	Lane 2	Lane 1	Lane 2
10:00∼10:10	23	63	86	21	63	84	−8.70	0.00	−2.33	1	3	0	2	0
10:10∼10:20	35	62	97	34	63	97	−2.86	1.61	0.00	1	1	0	1	1
10:20∼10:30	25	59	84	23	59	82	−8.00	0.00	−2.38	2	1	0	1	0
10:30∼10:40	19	49	68	18	49	67	−5.26	0.00	−1.47	2	0	0	1	0
10:40∼10:50	29	72	101	27	72	99	−6.90	0.00	−1.98	2	1	0	1	0
10:50∼11:00	26	60	86	24	61	85	−7.69	1.67	−1.16	2	1	0	1	1
Total	157	365	522	147	367	514	−6.37	0.55	−1.53	10	7	0	7	2

## References

[1] Gordon R.L., Reiss R.A., Haenel H., Case E.R., French R.L., Mohaddes A., Wolcott R. (1996). Traffic Control Systems Handbook.

[2] McGowen P., Sanderson M. Accuracy of Pneumatic Road Tube Counters.

[3] Mimbela L.E.Y., Klein L.A. (2001). A Summary of Vehicle Detection and Surveillance Technologies Used in Intelligent Transportation Systems.

[4] Wang J., Wu M. An Overview of Research on Weigh-in-Motion System.

[5] Duzdar A., Kompa G. Applications Using A Low-Cost Baseband Pulsed Microwave Radar Sensor.

[6] Kranig J., Minge E., Jones C. (1997). Field Test of Monitoring of Urban Vehicle Operations Using Non-Intrusive Technologies.

[7] Kastrinaki V., Zervakis M., Kalaitzakis K. (2003). A survey of video processing techniques for traffic applications. Image Vis. Comput..

[8] Ushio N., Shimizu T. Loop *vs.* ultrasonic in Chicago: Ultrasonic vehicle detector field test isolating diffused reflection and enduring harsh environment.

[9] Forren J.F., Jaarsma D. Traffic Monitoring by Tire Noise.

[10] Cheung S.Y., Varaiya P. (2007). Traffic Surveillance by Wireless Sensor Networks: Final Report.

[11] Ma W., Xing D., McKee A., Bajwa R., Flores C., Fuller B., Varaiya P. A. (2014). WirelessAccelerometer-Based Automatic Vehicle Classification Prototype Systems. IEEE Trans. Intell. Transp. Syst..

[12] Barbagli B., Manes G., Facchini R., Manes A. Acoustic Sensor Network for Vehicle Traffic Monitoring.

[13] Satish V.R., Sonal C. (2012). Management of Car Parking System Using Wireless Sensor Network. Int. J. Emerg. Technol. Adv. Eng..

[14] EngHan N., SuLim T., Jesus G.G. Road traffic monitoring using a wireless vehicle sensor network.

[15] Youngtae J., Jinsup C., Inbum J. (2014). Traffic Information Acquisition System with Ultrasonic Sensors in Wireless Sensor Networks. Int. J. Distrib. Sens. Netw..

[16] (1992). The Federal Highway Administration (FHWA). Effects of Raising and Lowering Speed Limits.

[17] MNDOT (Minnesota Department of Transportation) Available online: http://iris.dot.state.mn.us (accessed on 20 March 2014)s

[18] Crossbow Technology. http://www.xbow.com.

[19] Devantech Ltd. http://www.robot-electronics.co.uk.

[20] TinyOS Alliance. http://www.tinyos.net.

